# An engineered scorpion toxin analogue with improved Kv1.3 selectivity displays reduced conformational flexibility

**DOI:** 10.1038/srep18397

**Published:** 2015-12-22

**Authors:** Adam Bartok, Krisztina Fehér, Andrea Bodor, Kinga Rákosi, Gábor K. Tóth, Katalin E. Kövér, Gyorgy Panyi, Zoltan Varga

**Affiliations:** 1 Department of Biophysics and Cell Biology, University of Debrecen, Egyetem tér 1, Debrecen, H-4012, Hungary; 2 Department of Inorganic and Analytical Chemistry, University of Debrecen, Egyetem tér 1, Debrecen, H-4032, Hungary; 3 Department of Organic and Macromolecular Chemistry, Ghent University, Ghent, Belgium; 4 Laboratory of Structural Chemistry and Biology, Institute of Chemistry, Eötvös Loránd University, Pázmány Péter sétány 1/A, Budapest, H-1117, Hungary; 5 Department of Medical Chemistry, University of Szeged, Dóm tér 8, Szeged, H-6720, Hungary; 6 MTA-DE Cell Biology and Signaling Research Group, University of Debrecen, Debrecen, Egyetem tér 1, H-4032, Hungary; 7 MTA-DE-NAP B Ion Channel Structure-Function Research Group, RCMM, University of Debrecen, Debrecen, Egyetem tér 1, H-4032, Hungary

## Abstract

The voltage-gated Kv1.3 K^+^ channel plays a key role in the activation of T lymphocytes. Kv1.3 blockers selectively suppress immune responses mediated by effector memory T cells, which indicates the great potential of selective Kv1.3 inhibitors in the therapy of certain autoimmune diseases. Anuroctoxin (AnTx), a 35-amino-acid scorpion toxin is a high affinity blocker of Kv1.3, but also blocks Kv1.2 with similar potency. We designed and produced three AnTx variants: ([F32T]-AnTx, [N17A]-AnTx, [N17A/F32T]-AnTx) using solid-phase synthesis with the goal of improving the selectivity of the toxin for Kv1.3 over Kv1.2 while keeping the high affinity for Kv1.3. We used the patch-clamp technique to determine the blocking potency of the synthetic toxins on hKv1.3, mKv1.1, hKv1.2 and hKCa3.1 channels. Of the three variants [N17A/F32T]-AnTx maintained the high affinity of the natural peptide for Kv1.3 but became more than 16000-fold selective over Kv1.2. NMR data and molecular dynamics simulations suggest that the more rigid structure with restricted conformational space of the double substituted toxin compared to the flexible wild-type one is an important determinant of toxin selectivity. Our results provide the foundation for the possibility of the production and future therapeutic application of additional, even more selective toxins targeting various ion channels.

Several peptide toxins were isolated from animal venoms in the last decades, which are high affinity blockers of different ion channels including voltage-gated potassium channels^1^,^2^,^3^. Typically, peptide toxins plug the channel pore from the extracellular side, thereby inhibiting the ionic flux.

In human T-cells K^+^ channels contribute to the maintenance of the negative resting membrane potential and thus, to the regulation of Ca^2+^ signalling controlling T-cell activation. Kv1.3 is the predominant voltage-gated K^+^ channel of effector memory T cells^4^. Since Kv1.3 blockers persistently inhibit the activation and proliferation of these T cells, Kv1.3 has emerged as an attractive pharmacological target in the treatment of several T-cell mediated autoimmune diseases such as Multiple Sclerosis, Type I diabetes, and asthma^5^,^6^. Experiments with animal models of these diseases proved the efficacy of peptide blockers of Kv1.3 in improving clinical symptoms without being toxic or immunogenic even during prolonged systemic administration. In order to avoid cross reactivity with other ion channels during *in vivo* application, which carries the danger of undesired side effects (e.g. Kv1.3 inhibitors which also block Kv1.2 may interfere with the function of neurons^7^), the drug molecules must be able to differentiate even among channel proteins that have minute structural variations.

Anuroctoxin (AnTx, αKTx 6.12) is a peptide toxin of 35 amino acids with a molecular weight of 4082.8, stabilized by four disulphide bridges, which was isolated by our workgroup from the venom of the scorpion *Anuroctonus* phaiodactylus^8^. AnTx is a high affinity blocker of Kv1.3 (*K*_*d*_ = 0.73 nM), however, with lower affinity it also blocks Kv1.2 (*K*_*d*_ = 6.14 nM). Our aim was to improve the selectivity of the wild-type toxin for Kv1.3 by site-directed changes in the primary sequence of the peptide.

By virtue of their larger and more complex contact surface with the targeted channel, peptide blockers are generally significantly more selective and are effective at much lower concentrations than non-peptide small molecule blockers. Efforts to improve the selectivity of a natural toxin rely on information from sequence alignment of toxins with known activities, identification of interacting residues by mutant cycle analysis and docking simulations. These calculations generally assume fairly rigid structures with many spatial constraints on the position of interacting molecular entities. The comparison of amino acid sequences and the selectivity of toxins known to block Kv1.2 and Kv1.3 channels provided us with the information to design the substitutions in AnTx (Fig. 1).

Most K^+^ channel-blocking toxins share a characteristic structural motif, called Cysteine-Stabilized α/β motif, consisting of an α-helix connected to a β-sheet of at least 2 strands, (i.e. an αββ topology) stabilized by two disulphide bridges.

Many of these toxins contain a critically positioned pair of residues, often referred to as the “functional dyad” made up of the conserved lysine (K23 in AnTx) and an aromatic residue approximately 6–7 Å away (usually 9 positions downstream of the lysine, F32 in AnTx)^9^,^10^. The side chain of the critical lysine strongly interacts with the negatively charged selectivity filter of the channel^11^. The functional dyad was originally proposed to be necessary for high affinity block of Kv channels in general, but with more information available it seems to be critical for the high affinity block of Kv1.2, but not so much for Kv1.3. The aromatic dyad residue is a tyrosine in most toxins blocking Kv1.2 with high affinity, but the selectivity for Kv1.3 seems to benefit from the replacement of this tyrosine by other, more polar residues such as threonine or asparagine. Thus, we decided to first synthesize [F32T]-AnTx with the aim of improving selectivity for Kv1.3.

Another residue that appeared potentially important in selectivity based on sequence comparison and previous docking results was at the position corresponding to AnTx N17. This site is located between the α-helix and the first β-strand and thus does not interact with the channel at the pore entrance as the dyad residues do. This position is occupied by the positively charged arginine or the polar glutamine in all highly Kv1.2-selective toxins listed in the table. We therefore replaced the polar N17 residue by alanine in AnTx and generated [N17A]-AnTx, with the aim of reducing affinity for Kv1.2 and enhancing selectivity for Kv1.3. Additionally, we synthesized and characterized the N17A/F32T double substituted peptide with the expectation of generating a toxin with a possibly even more advantageous pharmacological profile.

Thus, based on conserved features of toxins selective for Kv1.3 or Kv1.2 we generated three toxin analogues: [F32T]-AnTx, [N17A]-AnTx, and the double substituted [N17A/F32T]-AnTx.

The [F32T]-AnTx substitution markedly improved toxin selectivity for Kv1.3, but with reduced affinity. The [N17A]-AnTx substitution had no significant effect alone, but the two substitutions in combination resulted in a high affinity and highly Kv1.3-selective toxin, [N17A/F32T]-AnTx. The structural and dynamic consequences of the substitutions were studied by NMR spectroscopy and molecular dynamics simulations. The differences observed in the conformational flexibility of the peptides suggest that the more rigid structure of the double substituted [N17A/F32T]-AnTx compared to that of the native toxin may be an important factor of toxin selectivity. In summary, here we propose a novel mechanism likely to influence toxin selectivity, which is based on the conformational dynamics of the peptide structure.

## Methods

### Peptide synthesis

Amino acids and coupling reagents for peptide synthesis were purchased from Orpegen (Heidelberg, Germany) and Bachem (Bubendorf, Switzerland). Resin for solid-phase synthesis was purchased from Varian (Shropshire, UK). Solvents and all other reagents were purchased from Sigma-Aldrich Kft. (Budapest, Hungary), and were used without further purification. The analytical high pressure liquid chromatography (HPLC) system was from Agilent (Waldbronn, Germany) with a Luna^®^ 5 μm C18(2) 100 Å column (4.6 × 250 mm) from Phenomenex (Torrance, CA, USA), the semi-preparative system was from Shimadzu (Kyoto, Japan) with a Luna^®^ 10 μm C18(2) 100 Å column (10 × 250 mm), also from Phenomenex. Mass spectrometry data was collected on a Finnigan TSQ 7000 (Waltham, MA, USA) instrument operating in positive electrospray ionization mode.

All peptides were synthesized with a CEM microwave peptide synthesizer (Matthews, NC, USA) by the solid-phase method applying 9-fluorenylmethyloxycarbonyl (Fmoc) chemistry. The peptides were synthesized on a 0.1 mmole scale using as solid support Fmoc-Lys(Boc)-Wang (0.36 mmol/g loading) resin. All amino acid side chains were protected by TFA-labile protecting groups: Asn(Trt), Arg(Pbf), Cys(Trt), Glu(OBu^t^), Gln(Trt), His(Trt), Lys(Boc) and Thr(Bu^t^), except for the *N*-terminal pyroglutamic acid used without protecting group. For each peptide synthesis, the resin was first swelled in DMF for 15 min. For deprotection of the *N*^*α*^-Fmoc protecting group 20% piperidine/80% DMF was applied. Elongation of the peptides used standard Fmoc SPPS chemistry with 4 equivalents (equivalents relative to peptide synthesis scale in mmoles) HBTU, 4 equivalents HOBt, 4 equiv. Fmoc-AA-OH, as activator base DIEA was applied. Stock solutions prepared for the synthesis were 0.5 M HBTU and HOBt in DMF, 0.2 M Fmoc-AA-OH in DMF and 2 M DIEA in NMP. The CEM method is detailed in Sup. Table 1A. Peptides were detached from the resin with a 83% trifluoroacetic acid (TFA)/10% water mixture containing 5% (m/V) dithiothreitol and 2% (V/V) triisopropylsilane at room temperature for 3 hours. The resin was removed by filtration and the cleavage cocktails were dripped into cold diethyl ether to precipitate the peptide. The precipitated peptides were collected by filtration, washed with diethyl ether and dried. The cyclization of the crude peptides was made by the oxidative folding method using stirring under air in NH_4_OAc (pH 8) buffer solution (peptide concentration was 1 mg/ml) for 24 hours. The cyclic peptide toxins were dried by lyophilisation and purified by semi-preparative HPLC. (see Supplementary Table S1 for details)

### High pressure liquid chromatography (HPLC)

The crude peptides were purified by semi-preparative RP-HPLC on a Shimadzu semi-preparative HPLC system with LC-20AD pumps, SPD-20A UV-Vis detector, using a solvent system of eluent–A 0.1% TFA/H_2_O; eluent-B: 0.1% TFA, 20% H_2_O/acetonitrile on a Phenomenex Luna^®^ 10 μm C18(2) 100 Å column (10 × 250 mm). Gradient elution with the following profile was used to elute peptides from the column: 0% to 30% B over 60 minutes at a flow rate of 2.0 ml/min. Peptide signals were detected at 220 nm. Purity of the peptides was proved by analytical RP-HPLC on a Phenomenex Luna^®^ 5 μm C18(2) 100 Å column (4.6 × 250 mm).

### Separation and activation of lymphocytes

Human peripheral lymphocytes were isolated from the blood of healthy volunteers with Ficoll-Hypaque density gradient centrifugation. Collected cells were washed twice with Ca^2+^- and Mg^2+^-free Hanks’ solution containing 25 mM HEPES buffer, pH 7.4. Cells were cultured for 2 to 5 days in 24-well culture plates in a 5% CO_2_ incubator at 37 °C, in RPMI 1640 medium supplemented with 10% fetal calf serum (Sigma-Aldrich), 100 μg/ml penicillin, 100 μg/ml streptomycin, and 2 mM L-glutamine (density, 0.5 × 10^6^ cells per ml). The culture medium also contained 5, 7.5 or 10 μg/ml phytohemagglutinin A (Sigma-Aldrich), to increase K^+^ channel expression.

### Heterologous expression of Kv1.1, Kv1.2 and KCa3.1 channels

mKv1.1 currents were measured on L929 cells stably expressing the channel. Cells were cultured as described earlier^12^. CHO cells were transiently transfected with pcDNA3 vector containing the hKv1.2 gene or with pEGFP-C1 vector coding the KCa3.1 gene^10^ using Lipofectamine 2000 (Invitrogen) reagent according to the manufacturer’s protocol. In case of Kv1.2 the pcDNA3 vector was co-transfected with the plasmid coding GFP in a ratio of 10:1 (channel plasmid:GFP). GFP-positive transfectants were identified with a Nikon TE2000U fluorescence microscope (Nikon, Tokyo, Japan) and were used for current recordings.

### Electrophysiology

Solutions for whole-cell and outside-out patch-clamp measurements contained in mM: (bath: 145 NaCl, 5 KCl, 1 MgCl_2_, 2.5 CaCl_2_, 5.5 glucose, 10 HEPES, 0.1 mg/ml BSA, pH 7.35) and (pipette-filling solution for Kv channels: 140 KF, 2 MgCl_2_, 1 CaCl_2_, 10 HEPES, 11 EGTA, pH 7.22; for KCa3.1 channels: 150 K-aspartate, 5 HEPES, 10 EGTA, 8.7 CaCl_2_, 2 MgCl_2_, pH 7.22). Pipettes had 3-5-MΩ resistance in the bath. Series resistance compensation up to 85% was used.

The remaining current fraction at a given toxin concentration is RF=I/I_0_, where I and I_0_ are current amplitudes in the presence and absence of the toxin, respectively. A 2-parameter Hill equation was used to fit the dose-response relationships: *RF* = *K*_*d*_^*H*^*/(K*_*d*_^*H*^*+[Tx]*^*H*^) where *K*_*d*_ is the dissociation constant, *H* is the Hill coefficient and *[Tx]* is the toxin concentration^13^. When indicated, single-point estimate of the *K*_*d*_ was obtained assuming *H* = 1.

### 3D structure determination with NMR spectroscopy

The samples used in the NMR experiments were prepared by dissolving 5.2 mg of sAnTx and 4 mg of [N17A/F32T]-AnTx in 275 μl H_2_O:D_2_O = 9:1 solution containing phosphate buffer at pH 5.7, 50 mM NaCl and 1 mM NaN_3_.

The experiments were performed at ^1^H resonance frequencies of 500 MHz and 700 MHz on Bruker AVANCE II and AVANCE III spectrometers, respectively at 303K. The ^1^H and ^13^C chemical shift scales were calibrated using the signals of internal DSS at 0.0 ppm for ^1^H and for ^13^C. The ^1^H resonances were assigned with standard homonuclear NMR techniques in the CCPNMR program^14^.

The distance constraints were obtained from a NOESY spectrum with 100 ms mixing time in H_2_O solution. The NOE intensities were evaluated from the volume of the crosspeaks and calibrated internally in CCPNMR to an average peak intensity followed up with a recalibration during structure calculation using spin diffusion correction^15^ in ARIA2.3^16^. For structure calculation CNS1.2^17^ was employed with a subsequent refinement in explicit water^18^.

Backbone dihedral angle restraints derived from secondary chemical shift information using a consensus between the results obtained with TALOS+^19^ and DANGLE^20^ were used. A total of 100 structures were calculated and an ensemble of 10 structures was generated using acceptance test selecting structures with no bond length, valence angle and NOE violations. The accepted structures were visualized in PyMol and analysed with Procheck^20^.

### Molecular Dynamics (MD) Simulations

All MD simulations were performed using AMBER version 14^21^ implemented on GPUs^22^,^23^. The AMBER ff99SB force field^24^ for the peptides and the TIP3P model^25^ for water were used. The cut-off used for non-bonded interactions was 8 Å for NPT and 9 Å for NVE, respectively. The particle-mesh Ewald^26^ procedure was used to describe long-range electrostatic interactions with maximal grid spacing of 1 Å. Periodic boundary conditions were applied with a truncated octahedron geometry. The SHAKE^27^ algorithm was used to keep the bond lengths of hydrogen atoms rigid allowing a time step of 2 fs to be used. Three MD simulations were carried out: a 100 ns and a 10 μs MD using an NPT ensemble and a 1 μs MD using an NVE ensemble. *For the NVE simulation*, first a minimization was performed for 10000 steps starting with steepest descent algorithm, which was switched to conjugate gradient algorithm after 100 steps. Then a constant pressure MD was carried out for 0.5 ns with isotropic position coupling using a Berendsen barostat to equilibrate system, during which the density stabilized at around ca. 1 g/cm^3^, and the temperature settled at 300 K. Then the pressure regulation is switched off and a classical constant total energy MD is carried out for 1 μs. The translational centre of mass motions was removed every 1000 steps. *For NTP simulations* first minimization in 2000 steps was performed switching from steepest descent to conjugate gradient algorithm after 1000 steps. Then a relaxation is carried out using constant total energy MD for 50 ps while increasing temperature from 0 K to 50 K. The system was further relaxed in a 1 ns long MD simulation using an NPT ensemble with isotropic position coupling using a Berendsen barostat while increasing temperature from 50K to 310K during 500 ps and keeping it at this temperature for 500 ps and allowing the density to stabilize at around ca. 1 g/cm^3^. During both relaxation steps backbone atoms were restrained with 4 kcal/mol/A force constant. Finally, by releasing the restraints, a production MD was carried out for 100 ns and 10 μs.

*Analysis* 50000 coordinate snapshots were saved for analysis in all cases. The trajectories were analysed with cpptraj^28^ and visualized in VMD^29^. The stability of the trajectories was analysed using the mdout_analyser.py^21^.

## Results

### Chemical synthesis of AnTx variants

**Figure 2:**
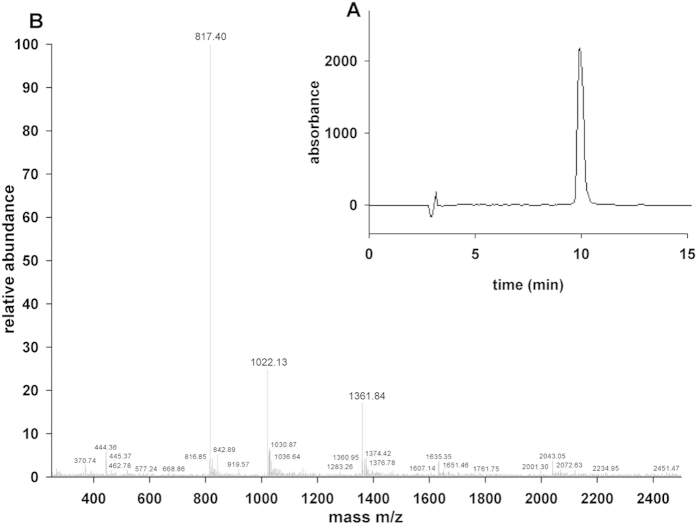
Chemical synthesis and purification of sAnTx. (**A**) Purity of synthetic AnTx samples was determined by HPLC as described in materials and methods using the parameters displayed in Supplementary Table S1. (**B**) Molecular weights of the peptides were determined and listed in Supplementary Table S1. The MS of sAnTx is shown.

The wild-type toxin and the designed mutants were generated by solid-phase chemical synthesis. Following chemical synthesis and folding the main components were finally purified using a C18 semi-preparative column (Phenomenex Luna) run with linear gradient from solvent A to 30% solvent B in 60 min. The structure and purity of the synthetic toxin were confirmed by analytical HPLC (Fig. 2A) and mass spectrometry (Fig. 2B). The retention times found by the analytical HPLC and the molecular mass of the peptides determined by mass spectrometry are displayed in Supplementary Table S1. The physiological effects of the synthetic wild type (WT) anuroctoxin were shown to be indistinguishable from that of the native peptide (see below).

### Electrophysiological characterization of AnTx variants

**Figure 3:**
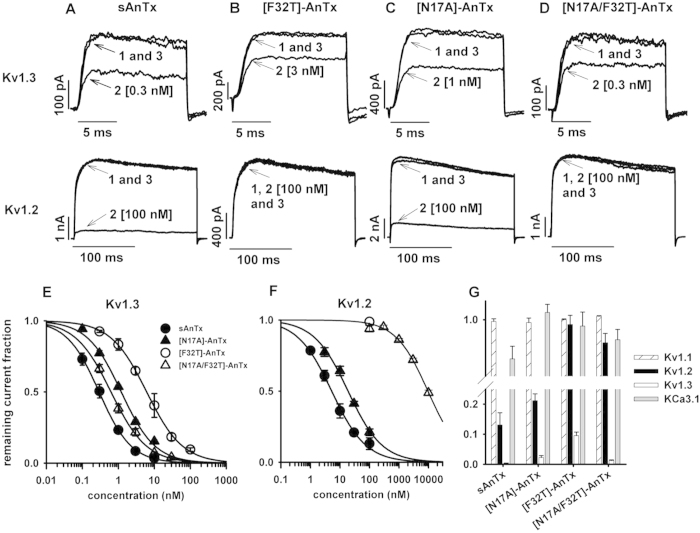
Selectivity profile of synthetic AnTx variants. The effect of (**A**) sAnTx, (**B**) [F32T]-AnTx, (**C**) [N17A]-AnTx and (**D**) [N17A/F32T]-AnTx on Kv1.3 and Kv1.2 channels. **Upper panels.** Kv1.3 currents were measured in activated human lymphocytes. The bath was perfused continuously. The traces show the K^+^ current before the application of the toxin (1), after the equilibration of the block in the presence of the indicated concentration of the toxin (2) and after recovery from block during the perfusion of the bath with toxin-free solution (3). **Lower panels.** Whole-cell Kv1.2 currents were measured in a transfected CHO cells. Traces are labelled as in the upper panels. See text for voltage protocols. Concentration-dependence of K^+^ current block by sAnTx (filled circles), [F32T]-AnTx (empty circles), [N17A]-AnTx (filled triangles) and [N17A/F32T]-AnTx (empty triangles) on (**E**) Kv1.3 and (**F**) Kv1.2 channels. The superimposed solid lines are the Hill equations fitted to the data points (see Material and Methods). The best fits yielded (**E**) *K*_*d*_ = 0.2 nM, and *H* = 1.0 for sAnTx, *K*_*d*_ = 6.2 nM and *H* = 1 for [F32T]-AnTx, *K*_*d*_ = 1.2 nM and *H* = 0.9 for [N17A]-AnTx and *K*_*d*_ = 0.6 nM and *H* = 0.9 for [N17A/F32T]-AnTx, and (**F**) *K*_*d*_ = 5.2 nM, and *H* = 0.8 for sAnTx, *K*_*d*_ = 20.0 nM and *H* = 0.8 for [N17A]-AnTx and *K*_*d*_ = 9.6 μM and *H* = 0.8 for [N17A/F32T]-AnTx. (**G**) Effect of AnTx variants at 100 nM on Kv1.1. Kv1.2, Kv1.3 and KCa3.1 channels (for details see Materials and Methods). The bars show the average remaining current fraction of 3–5 independent measurements at +50 mV where the error bars represent the S.E.M.

First we aimed at reproducing the effects of natural anuroctoxin with the wild-type toxin produced by solid-state chemical synthesis, denoted as sAnTx. The blocking effect of sAnTx was first tested on Kv1.3 channels expressed by activated human lymphocytes. Channels were activated by depolarizing pulses to +50 mV to maximize the open probability and pulse duration was limited to 15 ms to minimize inactivation. The use of such short pulses prevented cumulative inactivation and allowed us to record the current amplitude every 15 s giving better temporal resolution of the kinetics of block development and recovery. After reaching equilibrium block, 0.3 nM of sAnTx caused a significant reduction of the current amplitude (Fig. 3A, upper panel, trace 2; Fig. 3G) indicating that it had similar efficacy to the natural toxin. Perfusing the cell with toxin-free solution resulted in full recovery of the current (traces 1 and 3). Next the synthetic toxin was tested on Kv1.2 channels heterologously expressed by CHO cells. Due to the slower activation kinetics of Kv1.2, longer depolarizing pulses were applied, but because Kv1.2 also inactivates at a much slower rate than Kv1.3 no cumulative inactivation was induced even by the 200-ms-long pulses repeated every 15 s. Similarly to Kv1.3, Kv1.2 was also reversibly blocked by sAnTx (Fig. 3A, lower panel). By measuring the extent of block over a 100-fold concentration range for both channels we determined the dose-response relationships. The synthetic toxin (sAnTx) blocked Kv1.2 with a *K*_*d*_ = 5.2 nM (*H* = 0.8) and Kv1.3 with *K*_*d*_ = 0.2 nM (*H* = 1.0) compared to the respective values of 6.1 nM and 0.7 nM for the natural toxin (Fig. 3E,F)^8^. The good agreement of these values indicated that the synthetic toxin was functionally equivalent to the natural one, thus the synthesis and folding of the synthetic peptide were successful.

Next we tested the synthesized mutants on Kv1.3 and Kv1.2 channels. The applied concentration range was adjusted to the observed sensitivity of the channel to the toxin in order to obtain dose-response relationships.

Figure 3B, upper panel shows that 3 nM [F32T]-AnTx blocked ~40% of the Kv1.3 current which reduction is comparable to the effect of 0.3 nM sAnTx (see Fig. 3A, upper panel). Comparison of the *K*_*d*_ values shows that the F32T replacement reduced toxin affinity about 30-fold for Kv1.3 (*K*_*d*_ = 6.2 nM, *H* = 1.0, Fig. 3E) compared to sAnTx, but the reduction was much more pronounced for Kv1.2: at 100 nM concentration very small amount of block was detected (Fig. 3B, lower panel and 3F), the estimated increase in *K*_*d*_ was ~1000-fold. Thus, the expected improvement in selectivity for Kv1.3 was achieved, but was accompanied by a decrease in affinity. Figure 3C,E,F show that [N17A]-AnTx induces significant block of Kv1.3 and Kv1.2 currents, respectively, although the toxin is more potent on Kv1.3. The analysis of the dose-response relations show that [N17A]-AnTx substitution alone showed no improved selectivity over sAnTx: the affinity for both Kv1.3 and Kv1.2 decreased slightly (*K*_*d*_ = 1.2 nM, *H* = 0.9, Fig. 2E and *K*_*d*_ = 20.0 nM, *H* = 0.8, Fig. 3F, respectively) resulting in an approximate 17-fold selectivity for Kv1.3, which is inferior to that of the sAnTx. Fig. 3D upper panel shows that the [N17A/F32T]-AnTx double substituted peptide blocks the Kv1.3 current in sub-nanomolar concentration, the peptide at 0.3 nM concentration blocked approx. 33% of the whole-cell current whereas [N17A/F32T]-AnTx at 100 nM concentration induced a negligible block of the Kv1.2 current (Fig. 3D, lower panel). Analysis of the dose-response relationships shows that the [N17A/F32T]-AnTx double substitution resulted in a toxin with an affinity for Kv1.3 resembling that of the natural toxin (*K*_*d*_ = 0.6 nM, *H* = 0.9, Fig. 3E), but with an approximate 16000-fold selectivity over Kv1.2 (*K*_*d*_ = 9.6 μM, Fig. 3F) compared to the 9-fold selectivity of the natural toxin. The dose-response curves of sAnTx and its variants on Kv1.3 and Kv1.2 are summarized in Fig. 3E,F, respectively along with the remaining current fractions at 100 nM peptide concentrations (Fig. 3G).

**Figure 4:**
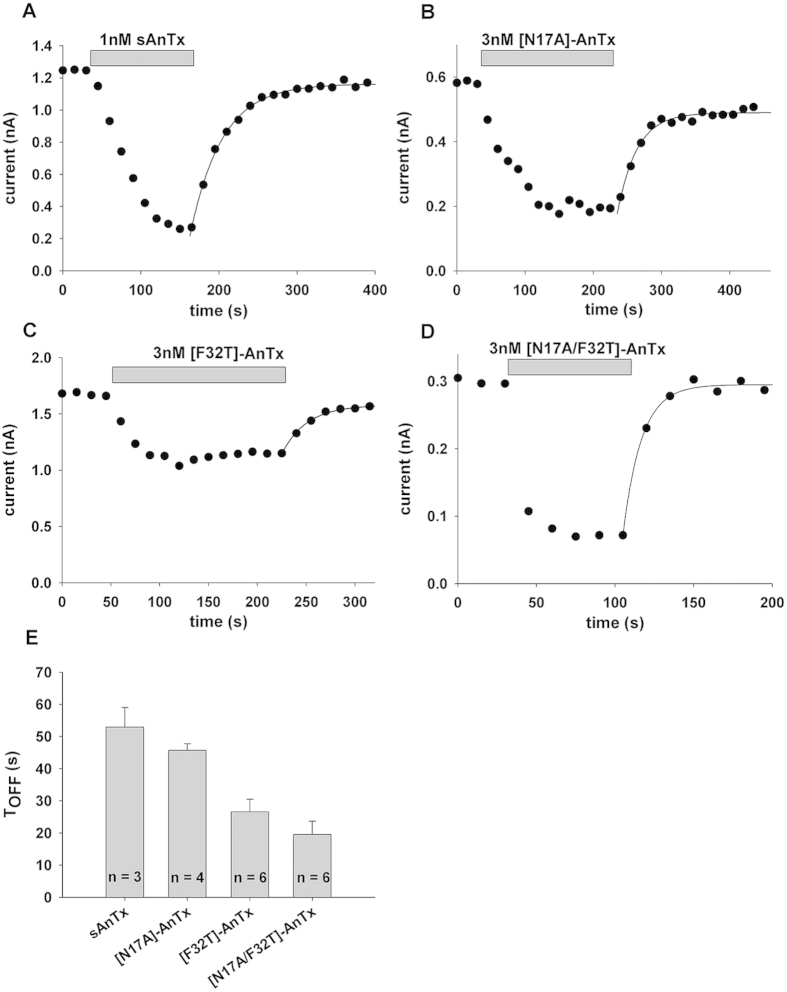
Time course of the development and the elimination of Kv1.3 current block by AnTx variants. Whole-cell Kv1.3 currents were evoked in human lymphocytes in response to depolarizing pulses to +50 mV from a holding potential of −100 mV every 15 s. Peak K^+^ currents were determined and plotted as a function of time. Grey bars mark the presence of an AnTx variant at the indicated concentration in the bath solution for sAnTx (1 nM, panel **A**,), [N17A]-AnTx (3 nM, panel **B**), [F32T]-AnTx (3 nM, panel **C**) and [N17A/F32T]-AnTx (3 nM, panel **D**). Superimposed solid lines indicate the best fit single exponential function to the data points obtained during the wash-out of the toxins: *A*(*t*) = *B* × (1 − exp(−*t/T*_*OFF*_)) + *C*, where *B* = *A*(*t* = ∞) − *C*, *A*(*t*) is the amplitude of the measured current at time *t, C* is the peak current at equilibrium block and *T*_*OFF*_ is the time constant for the elimination of the block upon perfusion of the bath with toxin-free solution. (**E)** Bars indicate the average *T*_*OFF*_ (±SEM) for the AnTx variants calculated from the indicated (n = 4–7) number of independent fits.

All observed blocking effects of the toxins on Kv1.2 and Kv1.3 were fully reversible. In order to gain more insight into the Kv1.3 - toxin interaction, wash-out kinetics of the sAnTx and the mutants were determined after reaching steady-state block (Fig. 4). Exponential fits to the current amplitudes yielded the following time constants (*T*_*OFF*_): 52.9 ± 6.1 s (n = 3) for sAnTx (Fig. 4A,E), 45.6 ± 2.0 s (n = 4) for N17A (Fig. 4B,E), 26.6 ± 4.1 s (n = 6) for [F32T]-AnTx (Fig. 4C,E) and 19.6 ± 2.83 s (n = 6) for [N17A/F32T]-AnTx (Fig. 4D,E), indicating that the dissociation rates were affected by the substitutions.

While the substitutions reduced toxin affinity toward Kv1.2 to various extents, they may have improved it for other channels that were not blocked by the natural toxin. To assess this possibility we tested the AnTx analogues on two other relevant channels: Kv1.1, the channel most closely related to the other two, and KCa3.1, the Ca^2+^ activated K^+^ channel, which plays an important role in the activation of naïve and central memory T cells. Supplementary Fig. S1 shows whole cell mKv1.1 and KCa3.1 currents in the absence and presence of 100 nM [N17A/F32T]-AnTx and following the wash-out of the peptide. The overlapping current records indicate the lack of significant blocking potency of [N17A/F32T]-AnTx on these channels. Neither sAnTx nor the single-substituted analogues caused significant reduction of the whole-cell Kv1.1 and KCa3.1 currents (Fig. 3G) at 100 nM concentration. These data indicate that the substitutions conserved the selectivity of the wild-type toxin for Kv1.3 over Kv1.1 and KCa3.1.

### NMR structures of sAnTx and [N17A/F32T]-AnTx

The ^15^N HSQC spectra of sAnTx and the double substituted [N17A/F32T]-AnTx showed well-resolved resonances as illustrated in Supplementary Fig. S2 and could be fully assigned. The amide resonances of T5 and K30 are broadened out and are shown in inlets drawn at lower threshold.

Slightly more distance restraints were obtained for sAnTx than for [N17A/F32T]-AnTx (575 versus 510), while the dihedral restraints were significantly less for the synthetic wild-type than for the double substituted peptide (38 versus 50). As a result, all residues have more than 10 restraints per residue to define their spatial arrangements yielding a well-determined structural ensemble with backbone RMSD of 1.27 Å and the heavy-atom RMSD of 1.87 Å for sAnTx and 0.85 Å backbone and 1.49 Å heavy-atom RMSD for [N17A/F32T]-AnTx. The main chain torsion angles of the final ensemble of 10 structures fall into the favoured and allowed regions of the Ramachandran plots (Supplementary Fig. S3) with 6.7% of the residues in the generously allowed regions for sAnTx as shown in Supplementary Table S2.

**Figure 5:**
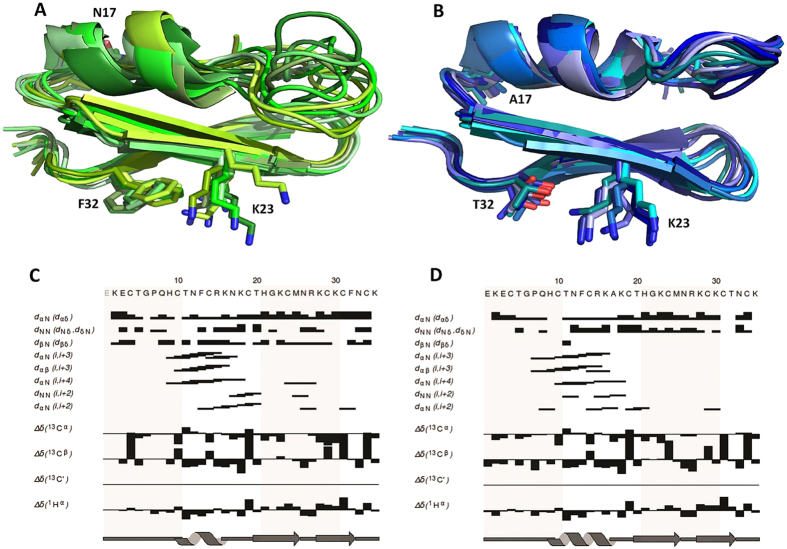
NMR structures of sAnTx and [N17A/F32T]-AnTx. NMR ensembles of 10 structures with no bond length, valence angle and NOE violations for (**A**) sAnTx and (**B**) [N17A/F32T]-AnTx. Important NOE contacts indicative of secondary structure and Secondary Chemical Shift charts for (**C**) sAnTx and (**D**) [N17A/F32T]-AnTx. The secondary chemical shift, defined as the difference between the observed and the random coil chemical shift, is dependent on secondary structure. In α-helices Cα atoms tend to have positive secondary chemical shifts, while for Hα and Cβ atoms mainly negative ones are observed. The pattern in β-strands is exactly opposite. Characteristic i,i+3 and i,i+4 NOE contacts can be seen at the position of the helix in both peptides.

The calculated NMR ensembles of sAnTx and its double substituted analogue are shown in Fig. 5A,B. Both peptides have characteristic Cysteine-Stabilized α/β-fold with secondary structures consisting of an α-helix and a double stranded antiparallel β sheet. Characteristic NOE contacts and secondary chemical shifts are shown in Fig. 5C,D. According to the DSSP algorithm^27^ as implemented in PyMOL, the helix extends between residues 10–16 in sAnTx with the core being between residues 11–15, while the β strands are between residues 20–25 and 28–32. The [N17A/F32T]-AnTx has a comparatively longer helix extending between 9-17 and β strands at the same position as in sAnTx.

**Figure 6:**
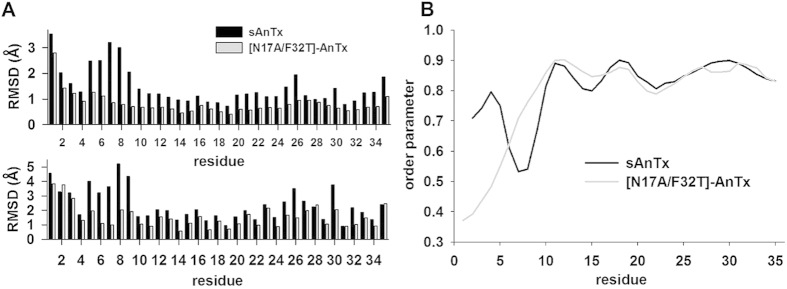
Structural comparison of sAnTx and [N17A/F32T]-AnTx. (**A**) Residue-wise RMSD of atomic coordinates along sAnTx and [N17A/F32T]-AnTx. Data are displayed for the backbone in the upper panel and for all heavy atoms in the lower panel for sAnTx (black bars) and [N17A/F32T]-AnTx (grey bars). The atomic coordinate RMSD values are calculated using the lowest energy structure as a reference. (**B**) Comparison of the per-residue order parameters (S^2^) derived from chemical shifts. The order parameter assumes a value between 0 and 1 with 1 corresponding to a perfectly rigid residue and 0 to a flexible residue.

The residue-wise RMSD of atomic coordinates (Fig. 6A) showed that the structures are well-determined at the positions of the secondary structure elements, while the loop connecting the N-terminal end and the α-helix between residues 6–9 is less well-defined in sAnTx than in [N17A/F32T]-AnTx.

## Discussion

In this study we aimed to design and synthesize Kv1.3-selective variants of a non-selective peptide. The applied substitutions were based on the comparison of the amino acid sequence of peptide toxins with known pharmacological profile.

We first targeted the essential dyad to improve selectivity. Both dyad residues proved critical for high affinity binding to Kv1.2 in Pi1 (K24 and Y33) and maurotoxin (MTX, K23 and Y32), two toxins preferring Kv1.2 over Kv1.3^30^,^31^. While the necessity of the dyad lysine was shown for other Kv channels as well^10^, the requirement for the aromatic half of the dyad, especially the tyrosine, is not as straightforward for blocking Kv1.3. Although block of Kv1.3 in the nanomolar range by toxins bearing a tyrosine at the aromatic dyad position is exemplified by charybdotoxin (ChTx^10^), Css20^13^, Tst26^32^, noxiustoxin^33^, hongotoxin-1^34^ and Pi1^30^, the selectivity for Kv1.3 seems to benefit from the replacement of this tyrosine by other residues. Many effective natural scorpion peptide inhibitors of Kv1.3 have a residue at the aromatic dyad position different from tyrosine such as phenylalanine (Pi2, Pi3, anuroctoxin), threonine (kaliotoxin, OSK1, BmKTX) or even asparagine (HsTx1), whereas a tyrosine is located at this position in toxins favouring Kv1.2 over Kv1.3 (MTX, Pi1, CoTX1, Pi4). This suggests that the presence of tyrosine at this position is rather disadvantageous if a Kv1.3 selective toxin is to be designed. The difference may stem from the presence of a histidine residue at position 399 in hKv1.3 at the external entryway of the pore, which prevents high affinity binding to a tyrosine. The equivalent V381 residue of hKv1.2 was shown to interact with the dyad Y32 of MTX, and the H399T replacement made Kv1.3 sensitive to MTX, while the V381H mutation in hKv1.2 drastically reduced MTX binding affinity^31^.

The phenylalanine found in AnTx has a similar aromatic nature as tyrosine, so the poor selectivity of this toxin for Kv1.3 is not surprising. The more polar side chains of threonine and asparagine appear to steer selectivity toward Kv1.3 over Kv1.2. Interestingly, the mutant [E16K,K20D]-OSK1 having T36 at the aromatic dyad position had somewhat lower Kv1.3 vs. Kv1.2 selectivity than its [E16K,K20D,T36Y]-OSK1 counterpart, implying that the outcome of the threonine/tyrosine exchange alone is not predictable. Nevertheless, we decided to first synthesize the [F32T]-AnTx with the aim of improving selectivity for Kv1.3.

The position corresponding to AnTx N17 is occupied by the positively charged arginine or the polar glutamine in all highly Kv1.2-selective toxins listed in the table. Docking simulations confirmed the importance of this residue: R19 of Pi4 and R14 of CoTx1 form salt bridges with negatively charged side chains of Kv1.2 residues and may aid in the initial positioning of the toxins^35^,^36^. We therefore replaced the polar N17 residue by alanine in AnTx and generated [N17A]-AnTx, with the aim of reducing affinity for Kv1.2 and enhancing selectivity for Kv1.3. Even though the effect of the double mutation cannot be reliably predicted from the effect of the single point mutations as exemplified by the OSK1 toxin^37^, we synthesized and characterized the N17A/F32T double substituted peptide with the expectation of generating a toxin with a possibly even more advantageous pharmacological profile.

Wild-type anuroctoxin and the three analogues were produced by chemical synthesis and their effect on mKv1.1, hKv1.2, hKv1.3 and hKCa3.1 channels was tested by patch-clamp. We have found that the F32T mutant lost the affinity for Kv1.2, however, its affinity for Kv1.3 also decreased slightly. The N17A substitution did not alter selectivity significantly. The double substitution N17A/F32T proved to be a high affinity and selective inhibitor of the Kv1.3 channel being an estimated 16000 times more selective over the other three channels tested.

Interestingly, the WT and N17A toxins, which block both Kv1.2 and Kv1.3 dissociate from Kv1.3 more slowly than the selective F32T and N17A/F32T substituted peptides as indicated by the wash-out time constants. This suggests that F32 forms favourable close contact interactions even with Kv1.3 channel residues around the pore that increase the residency time of the toxin. The F32T substitution abolishes this interaction as evidenced by the lower affinity and faster dissociation rate. The additional N17A substitution restores the high affinity of the toxin, but it dissociates from the channel at a much higher rate than the WT, which suggests that the mode of interaction of [N17A/F32T]-AnTx may be different from that of the WT with the channel^38^,^39^. Thus, we investigated the possible structural changes that may explain the different interactions by NMR and MD simulations.

Structural comparison of the synthetic wild type and the [N17A/F32T]-AnTx toxins based on solution state NMR structures showed that the two peptides have a very similar Cysteine-Stabilized α/β-fold, however, the structure of the double-substituted analogue is more defined: the overall backbone RMSD (1.27 Å versus 0.85 Å) as well as the heavy atom RMSD (1.87 Å versus 1.49 Å) are larger for sAnTx than for [N17A/F32T]-AnTx. Since comparable number of geometrical restraints was collected to calculate the sAnTx ensemble as for [N17A/F32T]-AnTx (an average of 16 distance restraints per residue in addition to 38 dihedral restraints), it appears that the structure of synthetic wild-type AnTx is intrinsically less defined than that of its double substituted variant. The dihedral angle restraint data based on Secondary Chemical Shifts indicated less ordered secondary structure elements for sAnTx than for N17A/F32T-AnTx. The order parameters (S^2^) derived from chemical shifts^40^ (Fig. 6B) indicate differences in the dynamics primarily in the N-terminal. Residues 6–9 are considerably less ordered in sAnTx than in [N17A/F32T]-AnTx. Indeed, these residues define a loop between the N-terminal and the helix at this position, which is highly undefined in the synthetic wild-type and very well-ordered in the double substituted peptide. Also, the chemical shifts indicate more order in the very N-terminal part of the wild-type peptide than in [N17A/F32T]-AnTx, this difference, however, is not reproduced in the structural ensembles.

Residue-wise RMSDs of the NMR ensembles (Fig. 6A) also indicate that a large fraction of the structural uncertainty in sAnTx is located at the loop between the N- terminal and the first helical element (residues 5–9), but there are elevated RMSD values along the whole sequence regarding the backbone (upper panel) as well as the side chain heavy atoms (lower panel). Specifically, the second β strand between residues 20–26, which is in direct contact with the K^+^ channel surface upon the insertion of K23 into the pore, displays higher backbone RMSDs of the atomic coordinates. This difference in the degree of definition of the structures suggests a hypothesis: due to its increased flexibility, the wild-type sAnTx can accommodate conformations suitable for unselective binding to both the Kv1.2 as well as to the Kv1.3 channels. On the other hand, due to the relative rigidity of structure, [N17A/F32T]-AnTx favours selective binding to Kv1.3, while it is less likely to adopt conformations suitable for binding to Kv1.2.

In order to test this hypothesis regarding the structural flexibility of the two peptides, we carried out multiple MD simulations with lengths of 100 ns, 1 μs and 10 μs for both sAnTx and [N17A/F32T]-AnTx.

**Figure 7:**
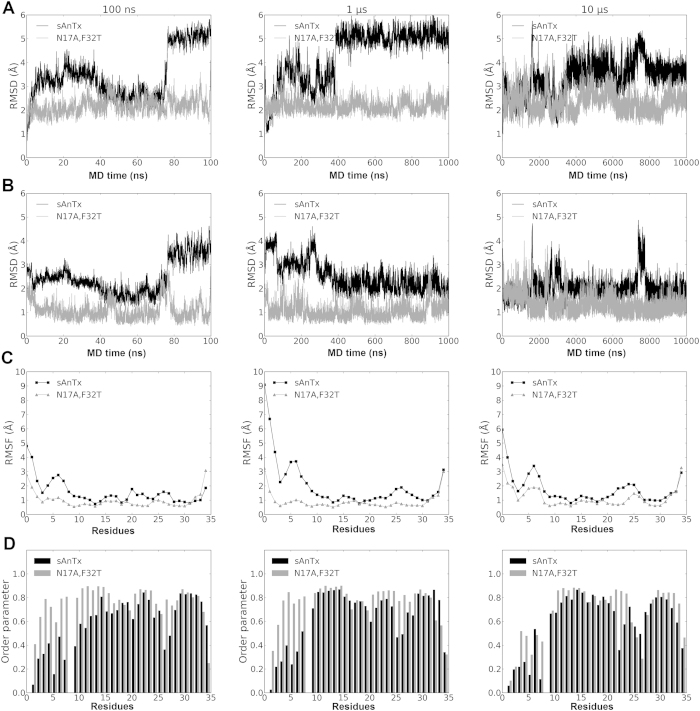
Comparison of sAnTx and [N17A/F32T]-AnTx in the three MD simulations of 100 ns, 1 μs and 10 μs lengths. Data are presented for sAnTx (black line) and for [N17A/F32T]-AnTx (grey line). The RMSD of structures calculated on the backbone atoms with respect to the (**A**) first and (**B**) an averaged structure over the trajectories. (**C**) Fluctuations in the atomic positions and order parameters of the NH bond vectors during the trajectories. The fluctuations were calculated for sAnTx (black line) and [N17A/F32T]-AnTx (grey line) with reference to the starting structure of the simulation. The fluctuations are mass-weighted average of atomic fluctuations of each atom for each residues. (**D**) Order parameters of the NH bond vectors.

RMSDs of atomic coordinates of the backbone heavy atoms with respect to the starting (Fig. 7A) and the average (Fig. 7B) structures along the trajectories show that the conformation of [N17A/F32T]-AnTx is more stable during the trajectory with consistently smaller deviations from the mean than that of sAnTx (standard deviation of the RMSDs from the means are in supplementary Table 2). Clearly, sAnTx is sampling more diverse conformations during the simulations. The single substituted [F32T]-AnTx demonstrated deviations whose magnitude was between those of the other two toxins (Supplementary Fig. S4).

We examined the positional fluctuation of positions (RMSF) of the backbone atoms on a per residue basis along the trajectory as shown in Fig. 7C. Increased fluctuations along the whole sequence are observed for AnTx in comparison with [N17A/F32T]-AnTx except for the very end of the C-terminal.

Furthermore, we extracted order parameters for the NH bond vectors of each residue using the Isotropic Reorientational Eigenmode Dynamics (iRED)^41^ analysis as implemented in cpptraj^28^. Order parameters characterize the dynamic processes taking place on the ps-ns timescale. The order parameters for AnTx as shown in Fig. 7D were consistently lower than that of its double mutant. These data further confirm our hypothesis that AnTx has higher structural flexibility than [N17A/F32T]-AnTx.

**Figure 8:**
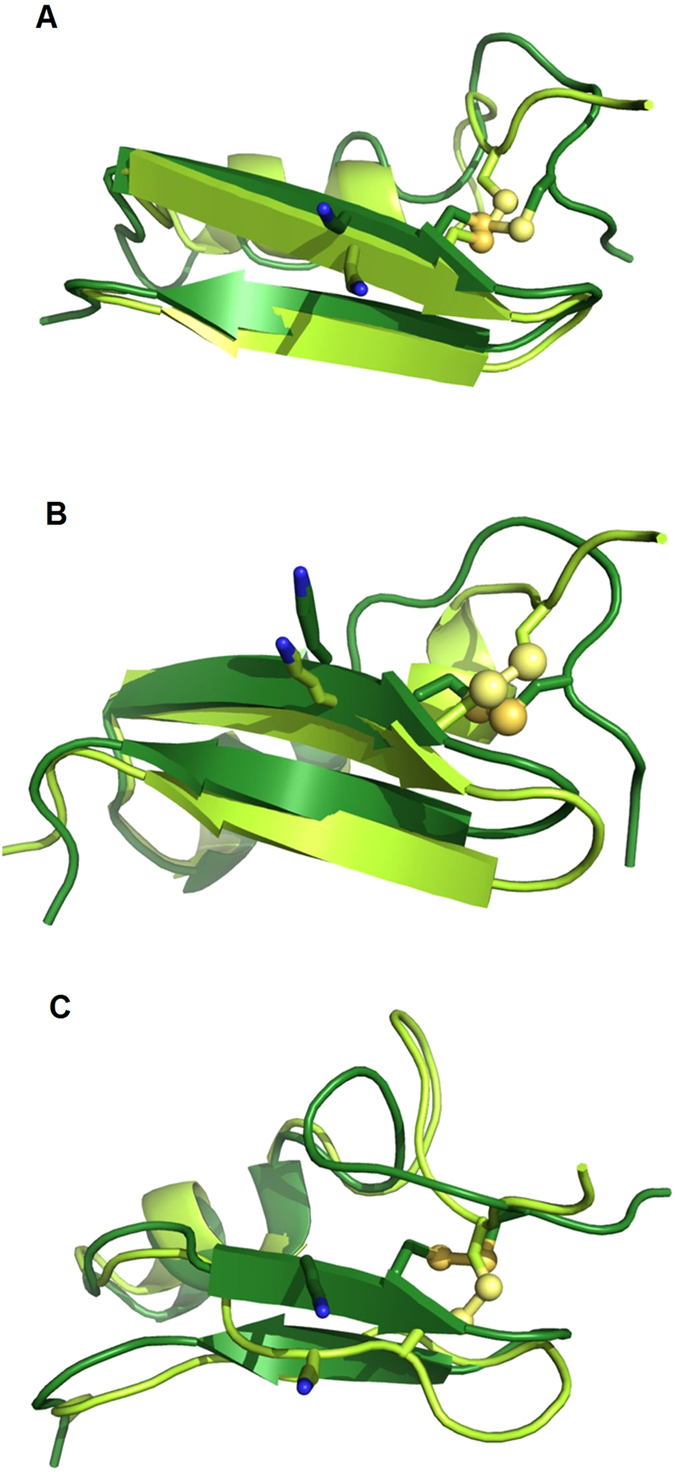
Overlay of the averaged conformations of sAnTx observed in the simulations showing the movement of the N terminal and the N terminal loop around the C4-C24 disulphide bond and its effect on the conformation of the β strand connected via the bridge. (**A**) for 100 ns simulation, averaged between 40–75 ns (light green) and 80–100 ns (dark green) (**B**). 1 μs, averaged between 0.5–2 μs (light green) and 4–10 μs (dark green), (**C**). 10 μs, averaged between 4–6 μs (light green) and 7.3–7.6 μs (dark green).

The atomic fluctuations and the order parameters indicate that the regions affected are the N terminal region between residues 1–10 and the loop between the β strands at residues 25–28. Visual inspection of the trajectory shows that the cause of the observed dynamics in sAnTx is the rearrangement of the entire N terminal preceding the helical segment: residues 5–9 and 1–3 are rotating around residue 4, which is anchored to the residue 24 on the first β strand via a disulphide bond (Fig. 8). The motions resulting from the rearrangements on the N terminal are transferred to the first β strand through the 4–24 disulphide bond and so influence the overall shape of the β sheet, particularly that of the first β strand containing the pore penetrating K23. Thus these rearrangements in sAnTx produce a dynamic ensemble, while [N17A/F32T]-AnTx is locked in a less diverse set of conformations. We observed a faster association rate for [N17A/F32T]-AnTx to Kv1.3 than for sAnTx, which we interpret such that the multiple conformations of sAnTx due to its higher flexibility slow the formation of the encounter complex from which the stable bound state develops^39^, whereas the more defined structure of [N17A/F32T]-AnTx allows faster docking (Supplementary Fig. S5).

The presence of the line-broadening of crosspeaks in the ^15^N HSQC spectrum indicates that there might be additional dynamic processes in the μs-ms timescale present for both AnTx and its double mutant, which are not sampled by the present MD simulations.

In summary, we have designed and synthesized three analogues of AnTx in order to improve the selectivity of the natural peptide among K^+^ channels^42^,^43^. We have shown that among the three peptides the [N17A/F32T]-AnTx analogue has the best pharmacological profile characterized by very high affinity and selectivity for Kv1.3 over mKv1.1, hKv1.2, and hKCa3.1 channels. NMR structure determination reported significant flexibility (RMSD) of the synthetic wild-type peptide over [N17A/F32T]-AnTx especially in the loop between the N-terminal and the first helical element and in the second β strand of the peptide. In line with this, molecular dynamics simulation reported larger variations in the conformations of the synthetic wild-type peptide than in the more rigid [N17A/F32T]-AnTx during multiple simulations.

Based on these we propose that due to the greater flexibility coupled with larger conformational variability, the wild-type AnTx can accommodate conformations suitable for unselective binding to both Kv1.2 as well as Kv1.3 channels, whereas the more rigid double substituted analogue is less likely to adopt conformations suitable for binding to Kv1.2 and thus favours selective binding to Kv1.3. Our results highlight a novel putative mechanism for selectivity by conformational flexibility of the peptides, and thus, may allow us to convert non-selective peptides to selective ones by stabilizing one of the possible conformations. This strategy may lead to the development of therapeutically applicable peptides with better pharmacological profiles, such as [N17A/F32T]-AnTx.

## Additional Information

**How to cite this article**: Bartok, A. *et al.* An engineered scorpion toxin analogue with improved Kv1.3 selectivity displays reduced conformational flexibility. *Sci. Rep.* **5**, 18397; doi: 10.1038/srep18397 (2015).

## Supplementary Material

Supplementary Information

## Figures and Tables

**Figure 1:**
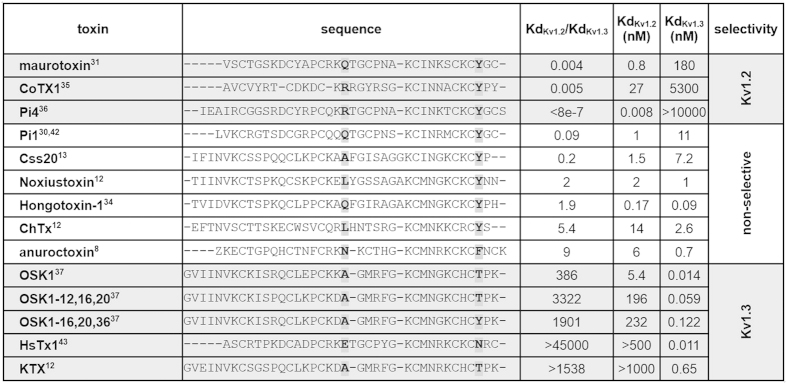
Sequence comparison of Kv1.2- and Kv1.3-selective toxins. The aligned primary sequences are shown for toxins for which the affinities for both Kv1.2 and Kv1.3 channels were determined. Toxins were classified as Kv1.2-selective, non-selective or Kv1.3-selective based on the ratio of the determined *Kd* values for the two channels. The positions of the two mutations (N17A and F32T) are indicated in the table in bold.
