# Supplementary material for: An engineered scorpion toxin analogue with improved Kv1.3 selectivity displays reduced conformational flexibility
Source: Sci Rep. 2015 Dec 22;5:18397. doi: 10.1038/srep18397 (PMC4686915; doi:10.1038/srep18397)
Supplement: Supplementary Information [file srep18397-s1.pdf]

An engineered scorpion toxin analogue with improved Kv1.3 selectivity displays reduced conformational flexibility

Adam Bartok[a,#], Krisztina Fehér[b,c,#,], Andrea Bodor[d], Kinga Rákosi[e], Gábor K. Tóth[e], Katalin E. Kövér[b,*], Gyorgy Panyi[a,f,*], Zoltan Varga[g,a]
[a]Department of Biophysics and Cell Biology, University of Debrecen, Egyetem tér 1, Debrecen, H-4012, Hungary

[b]Department of Inorganic and Analytical Chemistry, University of Debrecen, Egyetem tér 1, Debrecen, H-4032, Hungary

[c]Department of Organic and Macromolecular Chemistry, Ghent University, Ghent, Belgium

[d]Laboratory of Structural Chemistry and Biology, Institute of Chemistry, Eötvös Loránd University, Pázmány Péter sétány 1/A, Budapest, H-1117, Hungary

[e]Department of Medical Chemistry, University of Szeged, Dóm tér 8, Szeged, H-6720, Hungary

[f]MTA-DE Cell Biology and Signaling Research Group, University of Debrecen, Debrecen, Egyetem tér 1, H-4032, Hungary

[g]MTA-DE-NAP B Ion Channel Structure-Function Research Group, RCMM, University of Debrecen, Debrecen, Egyetem tér 1, H-4032, Hungary

[#] These authors contributed equally to the paper

*Corresponding authors:

Gyorgy Panyi
email: panyi@med.unideb.hu

Katalin E. Kövér
email: kover@science.unideb.hu

Supplementary Information

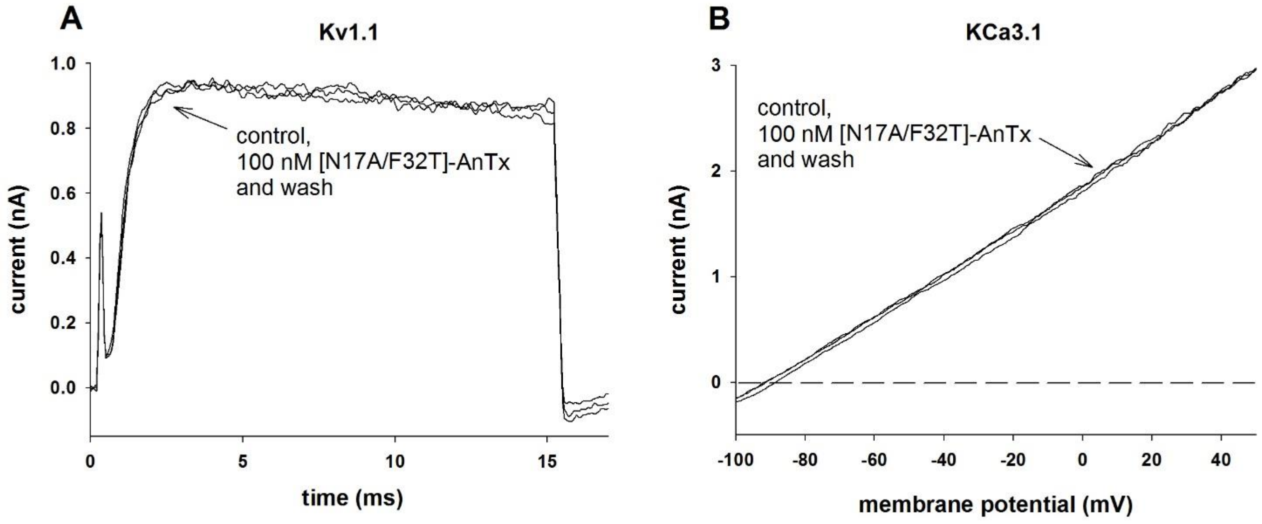

Supplementary Figure S1. [N17A/F32T]-AnTx is ineffective on Kv1.1 and KCa3.1 channels

A. Kv1.1 currents were measured in an L929 cell stably expressing the mKv1.1 channel. The bath was perfused continuously. Whole-cell currents were evoked by 15-ms-long depolarizing pulses to +50 mV from a holding potential of -100 mV every 15 s. The traces show the K^+ current before the application of the toxin (control), during the 20th episode in the presence of 100 nM [N17A/F32T]-AnTx and during the perfusion of the bath with toxin-free solution (wash). **B.** Whole-cell KCa3.1 currents were measured in a CHO cell transiently transfected with the KCa3.1 gene. The bath was perfused continuously. Currents were evoked using 200-ms-long voltage ramps running from -100 to +50 mV in order to distinguish KCa3.1 current from leak based on the reversal potential. Voltage ramps were delivered every 15 s. The displayed traces were recorded before the application of the toxin (control), during the 20th episode in the presence of 100 nM [N17A/F32T]-AnTx and during the perfusion of the bath with toxin-free solution (wash). For further experimental details see Materials and Methods.

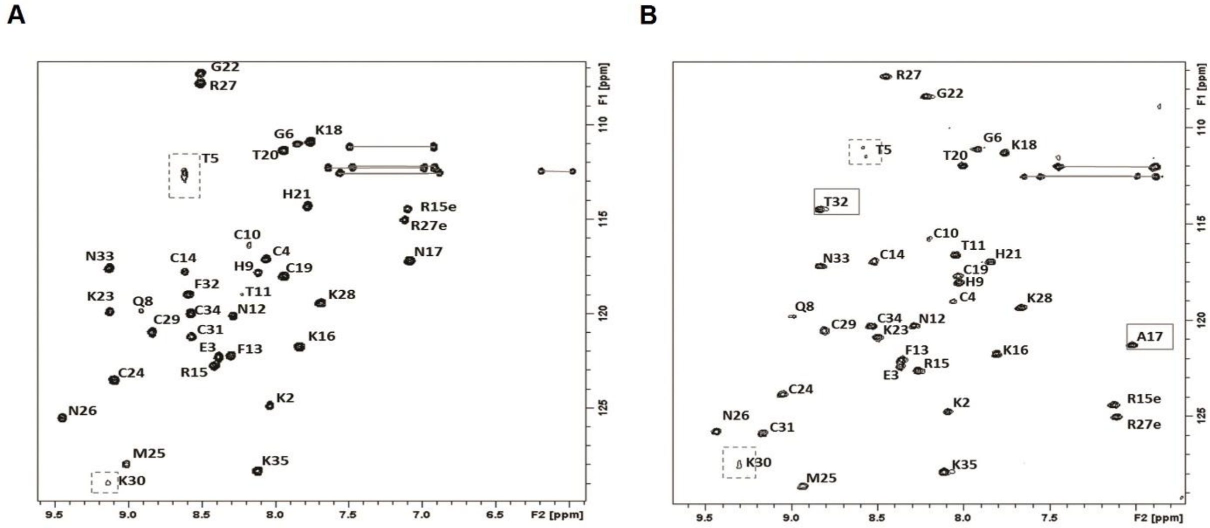

Supplementary Figure S2. ^{1}H-^{15}N HSQC spectra and assignments of the resonances.

Data are presented for **A**. sAnTx and **B.** [N17A/F32T]-AnTx. Inlets with dashed lines showing crosspeaks for T5 and K30 are drawn with lower threshold, side chain amide resonances are indicated by horizontal lines. The positions of substituted residues are indicated with a rectangle.

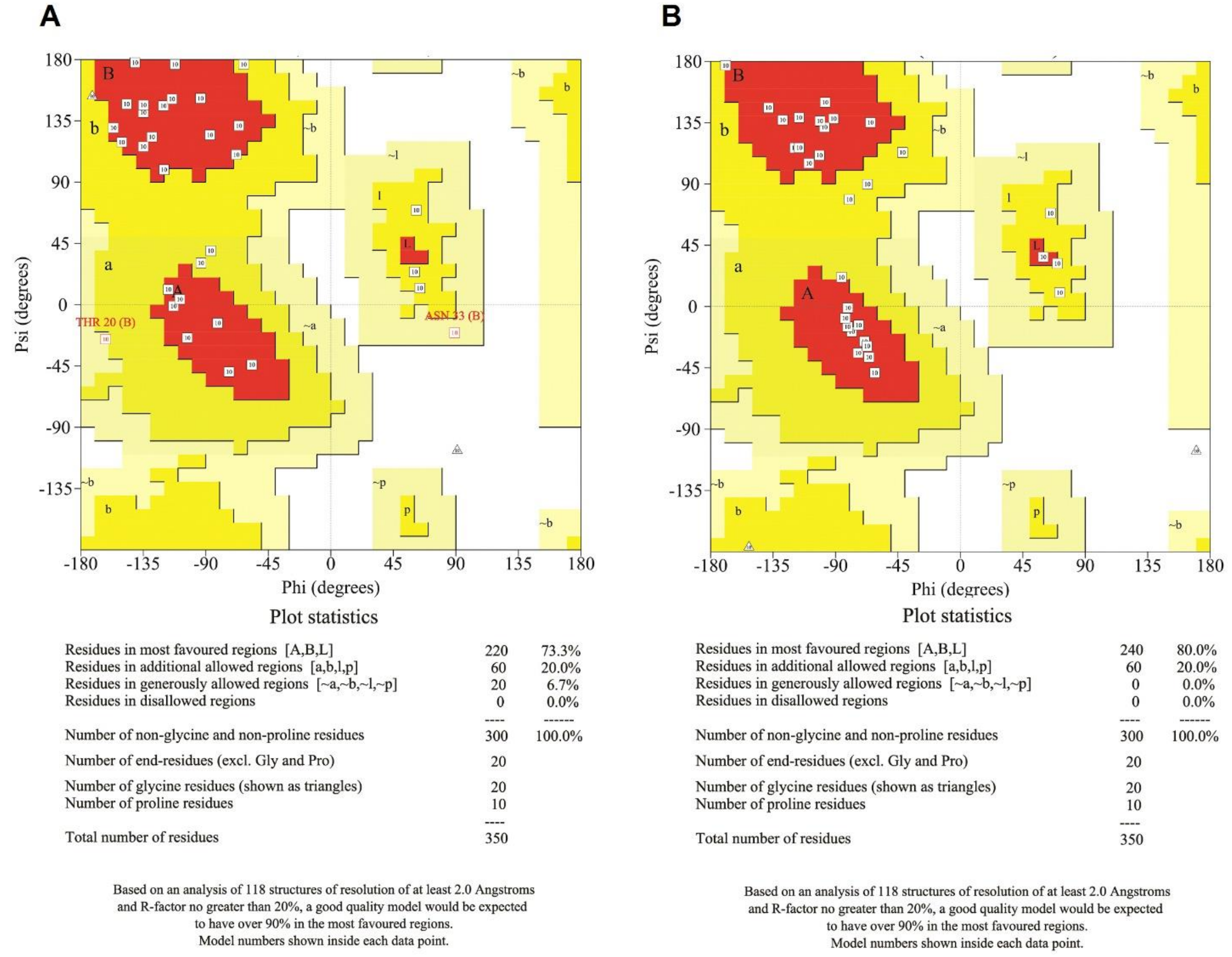

Supplementary Figure S3. Ramachandran plots

Data are presented for A) sAnTx, B) [N17A/F32T]-AnTx

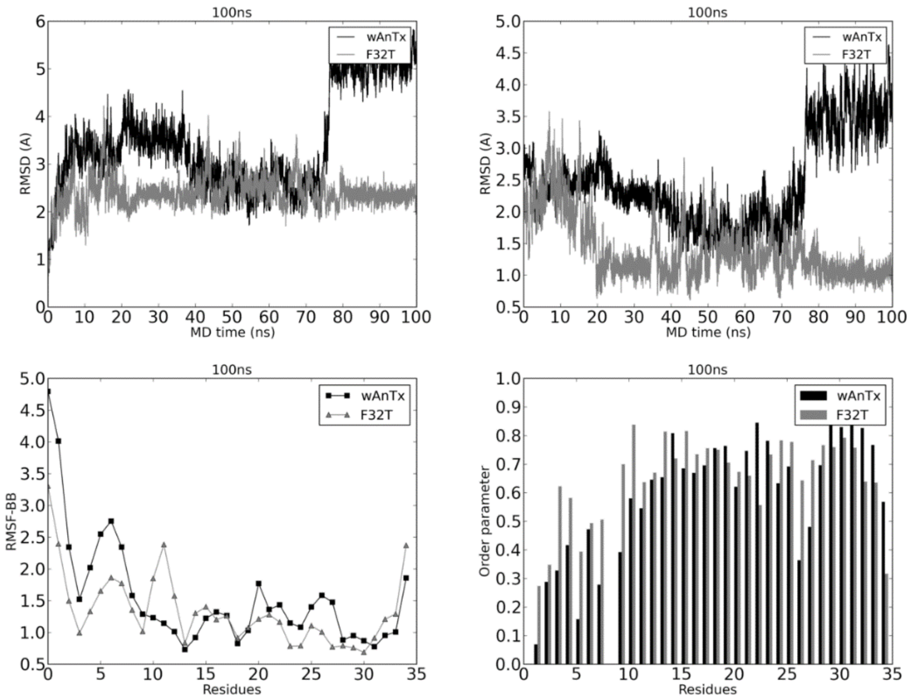

Supplementary Figure S4. 100 ns MD simulation of [F32T]-AnTx. Data are presented for sAnTx (black line) and for [F32T]-AnTx (grey line). The RMSD of structures calculated on the backbone atoms with respect to the first (top left) and an averaged (top right) structure over the trajectories. Fluctuations in the atomic positions (bottom left) and order parameters of the NH bond vectors (bottom right) during the trajectories. The fluctuations were calculated for sAnTx (black line) and [F32T]-AnTx (grey line) with reference to the starting structure of the simulation. The fluctuations are mass-weighted average of atomic fluctuations of each atom for each residue.

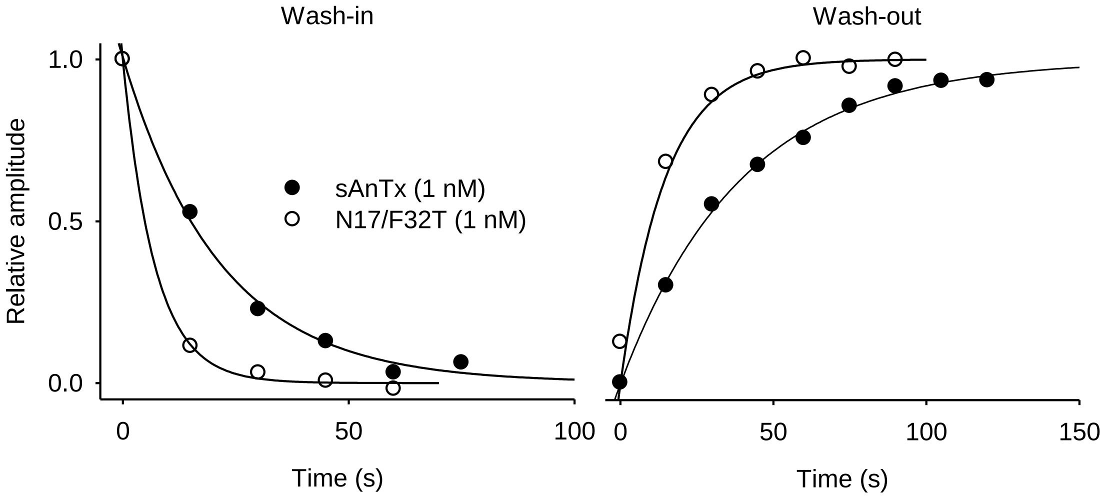

Supplementary Figure S5. Association rates of sAnTx and [N17A/F32T]-AnTx to Kv1.3
Normalized current amplitudes are plotted as a function of time during the development of block and recovery from block by the toxins. Depolarizing pulses were applied every 15 s. Data points were fit with single exponential functions and T_{ON} (wash-in) and T_{OFF} (wash-out) time constants were used to calculate k_{ON} values.
k_{ON} values characterizing the association rate were obtained from toxin wash-in kinetics at lower concentrations (at high concentrations block developed too quickly to be reliably fit) and wash-out kinetics (following high concentration block) on a cell-by-cell basis to exclude perfusion system positioning effects. k_{ON} was calculated as:

$$k_{ON} = \frac{1 - T_{ON} \times k_{OFF}}{T_{ON} \times [Tx]}, \qquad k_{OFF} = \frac{1}{T_{OFF}}$$

sAnTx had a significantly slower ON rate than [N17A/F32T]-AnTx despite its slightly higher affinity for Kv1.3 ($1.78 \pm 0.73 \times 10^7$ $M^{-1}s^{-1}$ vs $4.38 \pm 0.82 \times 10^7$ $M^{-1}s^{-1}$, respectively).

A

	Cycle	Nr. of cycles	Microwave power (W)	Max. Temperature (°C)	Reaction time (sec)
**Fmoc-deprotection**	single	1<br>1	35<br>40	75<br>75	30<br>180
**Coupling**	single	1	26	75	300
**Cys, Hys coupling**	double	2	0<br>25	50<br>50	120<br>240
**Arg coupling**	double	2	0<br>25	75<br>75	1500<br>30

B

Compound	Analytical HPLC grad.	t_R[a] (min)	$M_{calcd.}$	M_{found}[b] ($[M+H^+]$)
**sAnTx**	10-30 (%B); 20min	9.78	4083.79	4084.1
**[F23T]-AnTx**	10-25 (%B); 15min	8.58	4036.73	4036.9
**[N17A]-AnTx**	11-26 (%B); 15min	8.17	4040.76	4040.1
**[N17A/F32T]-AnTx**	10-25 (%B); 15min	10.09	3999.71	4000.2

Supplementary Table S1.

A. Optimized protocol for the CEM Liberty machine.

B. Analytical data of the peptide toxins.

[a]: Performed on an Agilent 1200 system (column: Phenomenex Luna 5 µm C18(2) 100Å; 250 x 4.6 mm; flow rate: 1.0 ml/min; eluent-A: 0.1% TFA/H_2O; Eluent-B: 0.1% TFA, 20% H_2O/acetonitrile) [b]: MS (ESI^+): *m/z*; Finnigan TSQ 7000 ESI-MS.

	AnTx	N17A/F32T
**Restraints**		
All	575	510
Intraresidual	335	274
Sequential	134	130
Medium range (2-3)	18	29
Long range (>3)	88	77
Ambiguous	72	105
Dihedral restraints	38	50
**Ramachandran plot (%)**		
favoured	73.3	80.0
allowed	20.0	20.0
generously allowed	6.7	0
disallowed	0	0
**RMSD of coordinates (Å)**		
backbone	1.27	0.85
heavy atom	1.87	1.49
all atom	2.32	1.96
**Average restraint violations**		
Bond	0.0076	0.0063
Angle	0.8195	0.5628
Distance	0.0426	0.0188
Dihedral	0.4851	0.1942

Supplementary Table S2. Structural statistics for sAnTx and [N17A/F32T]-AnTx.

The classification of NOE restraints is based on the proximity of the proton spins in the sequence. Ambiguous restraints are left assigned to multiple possibilities during the calculation. The Ramachandran plot regions are defined according to Morris et al.[1]. Violations are calculated using the following thresholds: 0.05 Å for bonds, 5° for valence angles, 0.5 Å for distances and 5° for dihedral angles.

1 Morris, A. L., MacArthur, M. W., Hutchinson, E. G. & Thornton, J. M. Stereochemical quality of protein structure coordinates. *Proteins* **12**, 345-364, doi:10.1002/prot.340120407 (1992).
